# Antibacterial and antibiotic-potentiation activity of the constituents from aerial part of *Donella welwitshii* (Sapotaceae) against multidrug resistant phenotypes

**DOI:** 10.1186/s12906-022-03673-3

**Published:** 2022-07-20

**Authors:** Michel-Gael F. Guefack, Marcelle O. Ngangoue, Armelle T. Mbaveng, Paul Nayim, Jenifer R. N. Kuete, Carine M. N. Ngaffo, Godloves F. Chi, Bathelemy Ngameni, Bonaventure T. Ngadjui, Victor Kuete

**Affiliations:** 1 Department of Biochemistry, Faculty of Science, University of Dschang, Dschang, Cameroon; 2 Department of Organic Chemistry, Faculty of Science University of Yaoundé 1, Yaoundé, Cameroon; 3 Department of Chemistry, Faculty of Science, University of Dschang, Dschang, Cameroon; 4 Department of Chemistry, Faculty of Science, University of Buea, Buea, Cameroon; 5 Department of Pharmacognosy and Pharmaceutical Chemistry, Faculty of Medicine and Biomedical Sciences, University of Yaoundé I, Yaoundé, Cameroon

**Keywords:** *Donella welwitschia*, Benzopehenone, Triterpenes, MDR bacteria, Antibacterial

## Abstract

**Background:**

The rise of multidrug-resistant (MDR) bacteria is a real public health problem worldwide and is responsible for the increase in hospital infections. *Donella welwitschii* is a liana or shrub belonging to the family Sapotaceae and traditionally used to cure coughs.

**Objective:**

This study was conducted with the objective to validate the medicinal properties of this plant, the aerial part was studied for its phytochemical composition using column and PTLC chromatography and exploring its antibacterial and antibiotic-modifying activity as well as those of its phytochemicals.

**Methods:**

The structures of the compounds were elucidated from their physical and spectroscopic data in conjunction with literature. The antibacterial activity of the isolated metabolites was performed toward a panel of MDR Gram negative and Gram-positive bacteria. The broth micro-dilution method was used to determine antibacterial activities, efflux pump effect using the efflux pump inhibitor (EPI) (phenylalanine-arginine-ß-naphthylamide (PAβN)), as well as the modulating activity of antibiotics. Monitoring the acidification of the bacterial growth medium was used to study the effects of the samples on the bacterial proton-ATPase pumps and cellular ATP production.

**Results:**

Eleven compounds were isolated including pentacyclic triterpenes, C-glucosyl benzophenones. With a MIC value < 10 μg/mL, diospyric acid (**7**) significantly inhibited the growth of *Escherichia coli* AG102, *Enterobacter aerogenes* ATCC13048, *Klebsiella pneumoniae* KP55, *Providencia stuartii* NEA16 and *Staphylococcus aureus* MRSA3. 28-hydroxy-*β*-amyrin (**8**) significantly impaired the growth of *Enterobacter aerogenes* EA27, *Klebsiella pneumoniae* ATCC11296 and *Staphylococcus aureus* MRSA6; and oleanolic acid (**9**) strongly impaired the growth of *Escherichia coli* AG 102, *Enterobacter aerogenes* EA27 and *Providencia stuartii* PS2636. Diospyric acid (**7**) and 28-hydroxy-*β*-amyrin (**8**) induced perturbation of H^+^-ATPase pump and inhibition of the cellular ATP production. Moreover, at MIC/2 and MIC/4, compounds **7**, **8**, and **9** strongly improved the antibacterial activity of norfloxacin, ciprofloxacin and doxycycline with antibiotic-modulating factors ranging between 2 and 64.

**Conclusion:**

The overall results of the current work demonstrate that diospyric acid (**7**), 28-hydroxy-*β*-amyrin (**8**) and oleanolic acid (**9**) are the major bioactive constituents of *Donella welwitschia* towards Gram-negative bacteria expressing MDR phenotypes.

**Supplementary Information:**

The online version contains supplementary material available at 10.1186/s12906-022-03673-3.

## Introduction

According to the World Health Organization (WHO), high levels of resistance to many pathogenic bacteria remains a serious health concern globally [1]. The rapid emergence of bacterial multidrug-resistant (MDR) phenotypes is a critical issue in the fight against infectious diseases [2]. Consequently, there is a need to search for new antimicrobial agents that can efficiently tackle bacterial resistance. The WHO has stated that most of the developing world still benefits from the use of traditional medicines derived from medicinal plants [3]. Moreover, many studies revealed the antibacterial inhibition activity of botanicals from African medicinal plants against MDR bacteria [4–9]. Several constituents including triterpenoids, phenolics, as well as alkaloids isolated from African medicinal plants were also documented for their antibacterial activities [10–13]. The present study focusses on the antibacterial activity of the constituents of *Donella welwitschii* (Engl.) Pierre ex Aubrev. & Pellegr. (Sapotaceae). The antibacterial activity of many secondary metabolites from plants belonging to the Sapotaceae family such as *Tridesmostemon omphalocarpoides* [14], *Omphalocarpum elatum* [15], *Chrysophyllum lacourtianum* [16], *Synsepalum msolo* [17], and *Manilkara zapota* [18] have been reported. The infusion of the shrub of *D. welwitschii* is traditionally used in the Central African Region and is used as an antitussive to relieve coughs and a stiff neck [19]. Previous phytochemical studies on species of the genus *Donella* reported cyclopropane type triterpene di-acids [20]. Herein, we reported the isolation of 11 secondary metabolites from *D. welwitschii*, and their antibacterial potential against a panel of MDR bacteria.

## Materials and methods

### Chemicals for antibacterial assays

A few reference antibiotics (RA), namely chloramphenicol (CHL), streptomycin (STR), erythromycin (ERY), norfloxacin (NOR), ciprofloxacin (CIP), ampicillin (AMP), and doxycycline (DOX) (Sigma-Aldrich, St. Quentin Fallavier, France) were predominantly used for the sample association tests, and only one was used as a positive control. Dimethyl sulfoxide (Sigma-Aldrich) was used to dissolve the samples before any tests. p-Iodonitrotetrazolium chloride (INT; Sigma-Aldrich) and phenylalanine-arginine-ß naphthylamide (PAβN; Sigma-Aldrich) were used as microbial growth indicator and efflux pump inhibitor (EPI), respectively. Solvents used for extraction and purification of bioactive compounds were of analytical grade.

### Plant materials and extraction procedure

**Fig. 1:**
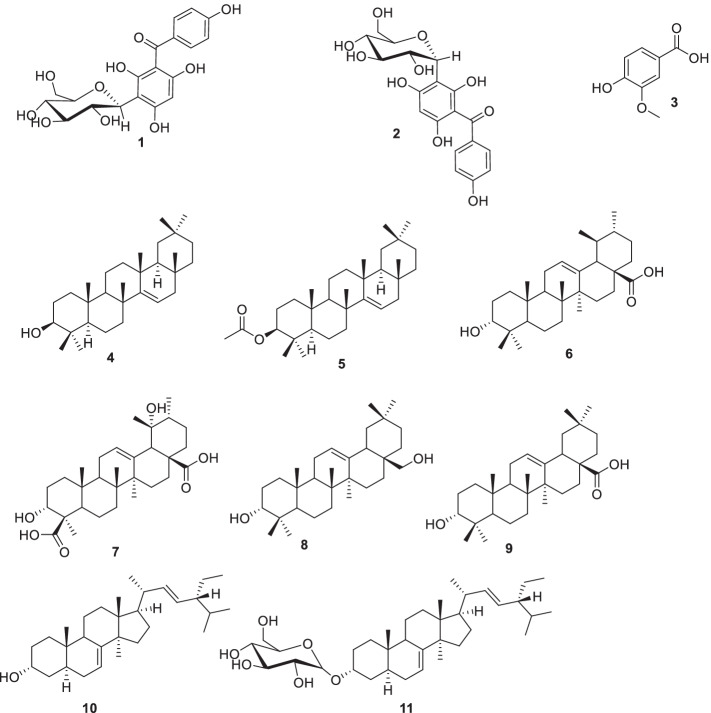
Compounds isolated from *D. welwitschia*. **1:** 3-*β*-_D_-glucopyranosyl-2,4,6-trihydroxyl (4-phenyl) methanone, **2:** 3-*α*-_D_-glucopyranosyl-2,4,6-trihydroxyl (4-phenyl) methanone, **3:** vanillic acid, **4:** taraxerol, **5:** taraxeryl acetate, **6:** ursolic acid, **7:** diospyric acid, **8:** 28-hydroxy-*β*-amyrin, **9:** oleanolic acid, **10:** spinasterol **11:** 3-*O*-*β*-_D_-glucopyranosyl spinasterol

The aerial part of *D. welwitschii* was collected from Manjo in Bertoua, the East region of Cameroun on May 2018. The appropriate authorisation has been obtained for the collection of the plant and its use has been carried out in accordance with the relevant guidelines. The identification of the plant was carried out by Dr. Tchiengue Barthelemy at the Cameroon National Herbarium (Yaoundé) where a voucher specimen was conserved under specimen No: 10708SFC / 56630HNC. The air-dried and powdered twig and leaf (1160 g) of *D. welwitschii* were macerated in a mixture of CH_2_Cl_2_ – MeOH (1:1, 5 L) for 48 h, three times. The filtrate was evaporated at 40 °C under vacuum to yield 42.7 g of dark brown extract. The structures of compounds **(**Fig. 1) were determined by means of modern spectroscopic techniques (NMR and MS) and comparison with available literature (**SM1**). All ^1^H and ^13^C NMR spectra and major chemical shifts of these compounds are shown in the supplementary file (**SM****1**).

### Separation and purification of extracts of *D. welwitschii*

Forty-gram (40.0 g) of the crude extract was partitioned by solid-liquid process into hexane (DWa 12.2 g), ethyl acetate (DWb 11.7 g), and methanol (DWc 25.6 g) extracts. Part of the methanol extract was suspended in distilled water (200 mL) and extracted with n-butanol, this yielded n-BuOH extract (DWd 10.5 g) and aqueous extract (DWe 9.2 g). DWa and DWb were combined based on their TLC profiles to give 23.5 g of combined extract (DWab). The combined hexane and ethyl acetate extracts (DWab, 23.5 g) adsorbed on 30 g silica gel was applied to column packed with silica gel 60 (63–43 um Merck, particle size between 0.043 and 0.063 mm in diameter and porosity 230–400 mesh ASTM) eluting with hexane and ethyl acetate, ethyl acetate and methanol under gradient conditions. 189 fractions of 150 mL each were collected and pooled based on their TLC profiles. Hex: frs 1–15, Hex-EtOAc (95:5) frs 16–33, Hex-EtOAc (90:10) frs 34–50, Hex-EtOAc (85:15) frs 51–70, Hex-EtOAc (80:20) frs 71–92, Hex-EtOAc (75:25) frs 93–117, Hex-EtOAc (70:30) frs 118–132, Hex-EtOAc (60:40) frs 133–148, Hex-EtOAc (25:75) frs 149–158, EtOAc frs 159–171, EtOAc (85:15) frs 172–184. Further purification of sub fractions 1–50 led to the compound taraxeryl acetate, **5** (23 mg); 51–117 yielded spinasterol **10** (80 mg) and taraxerol **3** (35 mg) Ursolic **6** (27 mg) and oleanolic **9** (17 mg) acids respectively. Diospyric acid **7** (45 mg), 28-hydroxyolean-12-ene **8** (23 mg) and 3-*O*-*β*-_D_-glucopyranosyl spinasterol **11** (65 mg) were isolated from subfractions 118–132, 133–148 and 149–171 by purification on silica gel columns. The n-BuOH fraction (DWd, 9.32 g) was adsorbed on 20 g of fine silica, dried and applied to silica gel column eluted with DCM and Methanol under gradient conditions. 150 subfractions of 150 mL each were collected and pooled based on their TLC profiles. Subfractions 1–20 and 97–120 were purified by preparatory TLC yielding vanillic acid **3** (3 mg), 3-*α*-_D_-glucopyranosyl-2,4,6-trihydroxyl (4-phenyl) methanone **2** (80 mg) and 3-*α*-_D_-glucopyranosyl-2,4,6-trihydroxyl (4-phenyl) methanone **1** (60 mg) respectively.

### Bacteria strains, culture media and growth conditions

Thirteen pathogenic strains consisting of Gram-negative and Gram-positive bacteria were recruited to measure the bacteriostatic and bactericidal power of the samples used in the study. Gram-negative bacteria included multidrug-resistant (MDR) isolates (laboratory collection) and reference strains of *Escherichia coli* (ATCC 10536, and AG102), *Enterobacter aerogenes* (ATCC 13048 and EA27), *Klebsiella pneumoniae* (ATCC 11296 andKP55), *Providencia stuartii* (ATCC 29916 and NEA16) and *Pseudomonas aeruginosa* (PA01 and PA124). Note that the clinical strains came from the laboratory collection of the UMR-MD1, University of Marseille, France. The Gram-positive bacterial strains were specifically *Staphylococcus aureus* including a reference strain obtained from the American Type Culture Collection (ATCC) (ATCC 25923), and a methicillin-resistant *S. aureus* isolate (MRSA3 and MRSA6) obtained from the culture collection of the Laboratory of Microbiology, Graduate School of Pharmaceutical Sciences, University of Tokyo, Japan, and was provided by Prof. Dr. Dzoyem of the University of Dschang [21, 22]. Susceptibility patterns of the tested bacteria are given in the Supplementary file (**Table S****1****, SM****2**).

### Evaluation of minimum inhibitory concentration (MIC) and minimum bactericidal concentration (MBC)

The ability of the isolated compounds to stop growth and kill bacteria was achieved through the determination of minimum inhibitory concentrations (MIC) and minimum bactericidal concentrations (MBC). This was possible using microdilution methods as described by Eloff (1998) [23] with some modifications and improvements [22, 24]. Briefly, series of dilutions of the samples were performed in a 96-well microplate after dissolving the samples in 10% dimethyl sulfoxide (DMSO)/Mueller Hinton broth (MHB). Then 100 μL of bacterial inoculum (2 × 10^6^ colony forming units (CFU)/mL) was added to each well. Chloramphenicol was used as a reference substance. The final concentrations ranged 4–512 μg/mL. Plates were then covered with a sterile plate sealer and incubated at 37 °C for 18 h. Revelation was done by adding 40 μL of INT (0.2 mg/mL) followed by incubation at 37 °C for 30 min. The MIC of each sample was defined as the lowest concentration of the sample that completely inhibited the growth of the bacteria. The MICs obtained were classified as follows: significant activity MIC < 100 μg/mL (MIC < 10 μg/mL for pure compounds), moderate activity 100 μg/ mL < MIC ≤625 μg/ mL (10 < MIC ≤100 μg/mL for pure compounds), weak activity MIC> 625 μg/mL (MIC> 100 μg/mL for pure compounds) [22, 25].

After MIC readings, minimum bactericidal concentrations (MBC) were determined by introducing 150 μL of MHB into a new 96-well microplate, after addition of 50 μL of the microplate contents, where no microbial growth was observed, and which did not receive INT during the MIC reading. The MBC of each sample was determined after 48 h of incubating at 37 °C, by adding 40 μL of 0.2 mg/mL INT, as previously described [22]. MBC was the lowest concentration of the samples, which did not produce a colour change after adding INT. Each experiment was performed in triplicate and repeated three times.

### Evaluation of the role of efflux pumps in the antibacterial activity of the samples

After testing the isolated compounds alone, the same compounds were put in the presence of PaβN (at 30 μg/mL) against 13 bacterial strains including five MDR phenotypes (*E. coli* AG102, *E. aerogenes* EA27, *K. pneumoniae* KP55, *P. stuartii* PS2636 and *S. aureus* MRSA3) as previously described [25, 26]. The activity improvement factor (AIF) or fold increase of the activity was determined as ratio of MIC _(sample alone)_/MIC _(sample + PAβN)_ [22]. Each assay was performed in duplicate and repeated three times.

### Assessment of the antibiotic-modifying activity of the samples

The combination assay was performed as previously described, with some modifications made during the procedure [4, 7]. The test was briefly performed by introducing 100 μL of MHB into the wells of a 96-well microplate, followed by the addition of 100 μL of the test antibiotics in the first well of each column and progressive dilution. 50 μL of solution of test compounds at sub-inhibitory concentrations (MIC/2 and MIC/4) were then introduced into the wells, followed by the addition of 50 μL of bacterial inoculum. The microplates were sealed and incubated for 18 h at 37 °C. MICs were determined using the INT as previously described. The antibiotic modulation factor (AMF), calculated as MIC _(antibiotic alone)_/− MIC _(antibiotic + compound)_, was used to express the modulating effects of compounds on antibiotics [5, 22, 27]. Each test was performed in duplicate and repeated three times.

### H + -ATPase-mediated proton pumping assay

The ability of compounds **7** and **8** to inhibit the H + −ATPase mediated proton pumps of *E. coli* AG102 was assessed by monitoring the acidification of the bacterial growth medium as previously described [28] with slight modifications [22]. Briefly, 100 mL of bacteria suspension was cultured in MHB for 18 h at 37 °C. The resulting culture was centrifuged at 3000 tr/min for 10 min at 4 °C. The pellet was first washed twice in distilled water, then in 50 mM KCl and suspended in 50 mL of 50 mM KCl. Then, the cell suspension was incubated overnight (18 h) at 4 °C for glucose starvation. In 4 mL of the cell medium, 0.5 mL of the tested samples (MIC/2, MIC, and 2 × MIC) were added, and pH adjusted to 6.8 with 1 M HCl or 0.1 M NaOH. Upon 10 min pre-incubation at 37 °C, medium acidification was initiated by adding 0.5 mL of 20% glucose, followed by pH measurement every 10 min for 1 h using a pH-meter. Tube containing MHB, inoculum, and DMSO was used as negative control. Each essay was performed in triplicates.

### Measurement of cellular ATP concentration

The ability of compounds **7** and **8** to inhibit the cellular ATP production of *E. coli* AG102 was assessed as previously described [28] with slight modifications. Briefly, 100 mL of bacterial culture (1:100 v/v) was grown in MHB culture medium for 18 h at 37 °C, followed by centrifugation (3500×g, 10 min). The pellet was washed twice with distilled water, then with 50 mM KCl and suspended in 50 mL of 50 mM KCl. The cell suspension (1.5–2 × 108 CFU/mL) was incubated overnight (18 h, 4 °C) for glucose starvation and then centrifuged. To 4 mL of the reaction medium, 0.5 mL of compounds **7** and **8** corresponding to MIC/2, MIC, and 2 × MIC was added, and the pH was adjusted to 6.4. After 10 min of pre-incubation under shaking at 37 °C, acidification of the medium was initiated after addition of 0.5 mL of 20% (w/v) glucose. The pH measurement was recorded every 20 min for a total period of 160 min. The experiment was conducted in the presence of DMSO (control) at a final concentration of 2.5%, to measure the degree of acidification of the external medium in the absence of compounds **7** and **8**. The experiment was performed in triplicate and repeated twice. The measured pH was used to plot the pH versus time curve [pH = f (time)]. Any inhibition of acidification of the medium in the presence of samples was attributed to an inhibitory effect of the H + -ATPase pumps.

### Statistical analysis

Data from each experimental group was expressed as mean *±* SD. One-way analysis of variance (ANOVA) followed by Dunnett’s post-hoc test for multiple comparisons were used for statistical analysis of data using GraphPad Prism Version 8.1.0 (GraphPad Software, CA, USA). Differences were considered significant at a probability level of 5% (*p* < 0.05), ** *p-values* < 0.01, and *** *p-values* < 0.001.

## Results

### Phytochemistry

Column chromatography of the major fractions afforded eleven compounds, which were identified as 3-*β*-_D_-glucopyranosyl-2,4,6-trihydroxyl (4-phenyl) methanone (**1**), 3-*α*-_D_-glucopyranosyl-2,4,6-trihydroxyl (4-phenyl) methanone (**2**), vanillic acid (**3**), taraxerol (**4**), taraxeryl acetate (**5**), ursolic acid (**6**), diospyric acid (**7**), 28-hydroxy-*β*-amyrin (**8**), oleanolic acid (**9**), spinasterol (**10**) and 3-*O*-*β*-_D_-glucopyranosyl spinasterol (**11**). These compounds include two benzophenones: **1** and **2**, one phenolic acid **3**, six triterpenoids **4–9**, one sterol **10**, and one sterol saponin **11**.

### MICs and MBCs of phytochemicals

The antibacterial activity of the isolated compounds **1–4** and **7–10** has been evaluated against *E. coli* (ATCC 10536, and AG102), *E. aerogenes* (ATCC 13048 and EA27), *K. pneumoniae* (KP55 and ATCC 11296), *P. stuartii* (ATCC 29916, and NEA16), *P. aeruginosa* (PA01 and PA124), and *S. aureus* (ATCC 25923 MRSA3 and MRSA6). The results obtained show that phytochemicals **5** and **11** exhibited weak activities against all strains tested. However, the other phytochemicals showed variable antibacterial activities depending on the bacterial strains tested, with MICs ranging from 4 to 512 μg/mL (Table 1). Compounds **7** showed significant antibacterial activities (MIC < 10 μg/mL) against *E. coli* AG102, *E. aerogenes* ATCC 13048, *K. pneumoniae* KP55, *P. stuartii* NEA16 and *S. aureus* MRSA3, compound **8** (against *E. aerogenes* EA27, *K. pneumoniae* ATCC 11296 and *S. aureus* MRSA6); and compound **9** (against *E. coli* AG 102, *E. aerogenes* EA27 and *P. stuartii* PS2636). In the MBC determinations, the active phytochemicals exhibited bactericidal activity against at least one bacterial strain tested. Overall, the MBCs of the phytochemicals ranged from 32 to 128 μg/mL (Table 1). A closer look at the MBC/MIC ratios indicated that the tested samples had mainly bacteriostatic effects (MBC/MIC > 4).

**Table 1:** MIC and MBC (μg/mL) of phytochemicals

Bacterial strains		Tested samples, MIC, and MBC values in μg/mL																			
		1		2		3		4		6		7		8		9		10		CHL	
		MIC	MBC	MIC	MBC	MIC	MBC	MIC	MBC	MIC	MBC	MIC	MBC	MIC	MBC	MIC	MBC	MIC	MBC	MIC	MBC
*E. coli*	ATCC10536	256	nd	512	nd	512	nd	128	2048	126	1024	64	512	32	512	64	256	128	nd	8	64
	AG102	256	nd	128	nd	512	nd	512	nd	16	128	**4**	128	16	512	**8**	128	128	nd	8	128
*E. aerogenes*	ATCC13048	128	nd	256	nd	256	nd	512	nd	128	512	**8**	128	64	256	16	512	256	nd	4	128
	EA27	512	nd	512	nd	512	nd	256	nd	32	256	32	512	**8**	1024	**8**	512	512	nd	8	64
*K. pneumoniae*	ATCC11296	256	nd	256	nd	256	nd	512	nd	16	128	32	256	**4**	512	64	256	128	nd	4	32
	KP55	256	nd	512	nd	512	nd	256	nd	16	128	**4**	64	128	512	32	128	512	nd	8	32
			nd		nd		nd		nd												
*P. stuartii*	PS2636	128	nd	512	nd	512	nd	256	nd	128	nd	32	256	64	256	**8**	512	128	nd	8	64
	NEA16	512	nd	512	nd	512	nd	128	2048	32	128	**8**	128	128	256	128	512	64	nd	8	128
*P. aeruginosa*	PA01	256	nd	512	nd	512	nd	256	nd	256	nd	32	512	128	512	64	256	128	nd	4	32
	PA124	256	nd	512	nd	512	nd	512	nd	256	nd	32	256	64	512	32	128	128	nd	4	64
*S. aureus*	ATCC25923	128	nd	128	nd	512	nd	128	2048	128	nd	32	512	64	256	128	512	512	nd	2	32
	MRSA3	512	nd	128	nd	512	nd	64	nd	512	nd	**8**	128	128	256	128	512	512	nd	4	32
	MRSA6	256	nd	128	nd	512	nd	256	nd	64	256	64	512	**8**	256	128	512	512	nd	4	128

***MIC*** Minimal inhibitory concentration, ***MBC*** Minimal bactericidal concentration, Values in bold indicate signifcant activity [25]. **1:** 3-*β* -_D_-glucopyranosyl-2,4,6-trihydroxyl (4-phenyl) methanone, **2:** 3-*a*-_D_-glucopyranosyl-2,4,6-trihydroxyl (4-phenyl) methanone, **7:** *Diospyric acid,* **8:** 28-hydroxy-*β*-amyrin, **9:** Oleanolic acid, **6:** Ursolic acid, **10:** Spinasterol, **4:** Taraxerol, **3:** Vanillic acid, CHL: chloramphenicol

### Effect of PAβN on the antibacterial activity of the tested samples

The susceptibility of *E. coli* (ATCC 10536, and AG102), *E. aerogenes* (ATCC 13048 and EA27), *K. pneumoniae* (KP55 and ATCC 11296), *P. stuartii* (ATCC 29916, and NEA16), *P. aeruginosa* (PA01 and PA124), and *S. aureus* (ATCC 25923 MRSA3 and MRSA6) to compounds and chloramphenicol in the presence of PAβN, was evaluated. Phytochemicals **6**, **7**, **8** and **9** in the presence of PaβN showed improved antibacterial activity (MIC reduction) against the tested bacteria by 2 to > 64 times. These isolated compounds were more effective against MDR bacteria and mainly against Gram-negative bacteria. Moreover, PAβN did not affect the activity of chloramphenicol against Gram-positive bacteria (*S. aureus:* ATCC 25923 and MRSA3) (Table 2).

**Table 2:** MIC (in μg/mL) of phytochemicals and chloramphenicol in combination of PAβN against a panel of 13 bacterial strains

Bacterial strains	Tested Samples, MIC values in the presence of absence of PaβN (in μg/mL)																			
	1		2		3		4		6		7		8		9		10		CHL	
	MIC (+PAβN)	R	MIC (+PAβN)	R	MIC (+PAβN)	R	MIC (+PAβN)	R	MIC (+PAβN)	R	MIC (+PAβN)	R	MIC (+PAβN)	R	MIC (+PAβN)	R	MIC (+PAβN)	R	MIC (+PAβN)	R
*E. coli*																				
ATCC10536	256 (8)	32	–	–	–	–	128 (4)	32	126 (8)	16	64 (2)	32	32 (0.5)	64	64 (2)	32	128 (4)	32	8 (0.125)	64
AG102	256 (16)	16	–	–	–	–	512 (16)	32	16 (1)	16	4 (0.125)	32	16 (0.5)	32	8 (0.5)	16	128 (8)	16	8 (0.125)	64
*E. aerogenes*																				
ATCC13048	128 (8)	16	256 (4)	4	256 (8)	32	512 (8)	64	128 (2)	64	8 (0.5)	16	64 (1)	64	16 (0.25)	64	–	–	4 (0.25)	16
EA27	512 (8)	4	512 (8)	8	512 (16)	32	256 (8)	32	32 (1)	32	32 (1)	32	8 (0.25)	32	8 (0.125)	64	–	–	8 (0.125)	64
*K. pneumoniae*																				
ATCC11296	256 (16)	8	256 (8)	4	256 (16)	16	–	–	16 (2)	8	32 (2)	16	4 (0.25)	16	64 (1)	64	128 (16)	8	4 (0.5)	8
KP55	256 (8)	8	512 (8)	4	512 (16)	32	–	–	16 (0.5)	32	4 (0.25)	16	128 (4)	32	32 (1)	32	512 (8)	64	8 (0.125)	64
*P. stuartii*																				
PS2636	128 (4)	4	512 (16)	2	512 (8)	64	256 (16)	16	128 (16)	8	32 (0.5)	64	64 (2)	32	8 (0.125)	64	128 (16)	8	8 (0.25)	32
NEA16	512 (16)	8	512 (32)	4	512 (16)	32	128 (16)	8	32 (2)	16	8 (0.25)	32	128 (2)	64	128 (8)	16	64 (4)	16	8 (0.125)	64
*P. aeruginosa*																				
PA01	–	–	–	2	–	–	–	–	256 (8)	32	32 (1)	32	128 (4)	32	64 (2)	32	128 (8)	16	4 (1)	4
PA124	–	–	–	2	–	–	–	–	256 (32)	8	32 (0.5)	64	64 (1)	64	32 (0.5)	64	128 (4)	32	4 (0.5)	8
*S. aureus*																				
ATCC25923	128 (4)	4	128 (16)	1	512 (8)	64	128 (8)	16	128 (8)	16	32 (1)	32	64 (2)	32	128 (2)	64	512 (8)	64	2 (2)	1
MRSA3	512 (8)	1	128 (8)	4	512 (16)	32	64 (2)	32	512 (16)	32	8 (0.125)	64	128 (2)	64	128 (8)	16	–	–	4 (4)	1
MRSA6	256 (8)	1	128 (8)	1	512 (16)	32	256 (16)	16	64 (4)	16	64 (2)	32	8 (0.25)	32	128 (4)	32	–	–	4 (4)	1

*MIC* Minimal inhibitory concentration, *PAßN* Phenylalanine arginyl β-Naphtylamide, *R* Ameliorating Factor: MIC sample alone/MIC sample + PAβN ratio (this means the factor which determines the improvement of the activity of samples by PAβN; the activity of a sample was improved when its AIF was > 2); (**−**): MIC > 512 μg/mL for compound

### Antibiotic-modulating activity of compounds

The modulatory activity of active phytochemicals (**7**, **8** and **9**) was investigated on six classical antibiotics including streptomycin (STR), erythromycin (ERY), norfloxacin (NOR), ciprofloxacin (CIP), ampicillin (AMP) and doxycycline (DOX). The overall results showed a considerable increase in the activity of the tested antibiotics. The best potentiating effects were recorded with NOR, CIP and DOX, in combination with phytochemicals **7**, **8** and **9** at MIC/2 and MIC/4. It was found that the overall activity enhancement factors (AEFs) obtained with the antibiotics ranged from 2 to 64 (Table 3, Table 4 and Table 5), indicating a 2 to 64 fold increase in antibacterial activity of the antibiotics. These activities were predominantly recorded on the MDR strain *E. aerogenes* EA-CM64. The reference strain *E. coli* ATCC 10536 was also sensitive to the same combination (Table 3, Table 4 and Table 5). Combination of the other antibiotics and the compounds associations has mainly presented indifference or antagonistic effects.

**Table 3:** MICs of antibiotics in association with phytochemical **7** against selected bacteria

Antibiotics	Concentrations of compound 7	Bacterial strain, MIC (μg/mL) and AMF (in bracket)					
		***E. coli***		***P. aeruginosa***		***K. pneumoniae***	
		ATCC 10536	AG102	PA01	PA124	ATCC 11296	KP55
STR	0	8	16	64	32	16	4
	MIC/2	**2(4)**	**1(16)**	**16(4)**	**16(2)**	**1(16)**	**0.25(16)**
	MIC/4	**4(2)**	**4(4)**	**32(2)**	32(1)	**2(8)**	**1(4)**
ERY	0	8	4	16	16	32	8
	MIC/2	**4(2)**	**0.25(16)**	**4(4)**	**8(2)**	**16(2)**	**4(2)**
	MIC/4	**4(2)**	**0.5(8)**	**8(2)**	**16(1)**	**16(2)**	8(1)
NOR	0	32	2	16	8	32	16
	MIC/2	**8(4)**	**0.25(8)**	**4(4)**	**2(4)**	**1(32)**	4(4)
	MIC/4	**16(2)**	**0.5(4)**	16(1)	**2(4**)	**4(8)**	4(4)
CIP	0	8	4	8	16	8	8
	MIC/2	**0.25(32)**	**1(4)**	**2(4)**	**1(16)**	**0.5(16)**	**0.125(64)**
	MIC/4	**1(8)**	**2(2)**	**2(4)**	**1(16)**	**0.5(16)**	**1(8)**
AMP	0	4	4	32	16	8	8
	MIC/2	**0.5(8)**	**2(2)**	**16(2)**	**4(4)**	8(1)	**4(2)**
	MIC/4	**0.5(8)**	**2(2)**	**16(2)**	**8(2)**	8(1)	**4(2)**
DOX	0	2	8	8	8	16	8
	MIC/2	**0.25(4)**	**0.5(16)**	**2(4)**	**0.5(16)**	**0.5(32)**	**2(4)**
	MIC/4	**0.25(4)**	**1(8)**	**4(2)**	**2(4)**	**0.5(32)**	**4(2)**

Antibiotics tested at 256 μg/mL [STR streptomycin, *ERY* Erythromycin, *NOR* Norfloxacin, *AMP* Ampicillin, *CIP* Ciprofloxacin, *DOX* Doxycycline, *CHL* Chloramphenicol, *TET* Tetracycline]. *MIC* Minimal Inhibitory Concentrations; Values in bold represent significant antibacterial activity and significant antibiotic-modulating factor (AMF). AMF ≥2 was set as the cut-off for biologically significance of antibiotic resistance modulating effects [27]

**Table 4:** MICs of antibiotics in association with phytochemical **8** against selected bacteria

Antibiotics	Concentrations of compound 8	Bacterial strain, MIC (μg/mL) and AMF (in bracket)					
		***E. coli***		***P. aeruginosa***		***K. pneumoniae***	
		ATCC10536	AG102	PA01	PA124	ATCC11296	KP55
STR	0	8	16	64	32	16	4
	MIC/2	**0.25(32)**	4(4)	**16(4)**	**16(2)**	**32(2)**	**2(2)**
	MIC/4	**0.5(16)**	**16(1)**	**32(2)**	32(1)	**32(2)**	**2(2)**
ERY	0	8	4	16	16	32	8
	MIC/2	**4(2)**	**0.5(8)**	**4(4)**	**8(2)**	**4(8)**	**4(2)**
	MIC/4	**4(2)**	**1(4)**	**8(2)**	**16(1)**	**16(2)**	8(1)
NOR	0	32	2	16	8	32	16
	MIC/2	**8(4)**	**0.25(8)**	**4(4)**	**2(4)**	**1(32)**	**4(4)**
	MIC/4	**8(4)**	**0.5(4)**	16(1)	**2(4**)	**4(8)**	**4(4)**
CIP	0	8	4	8	16	8	8
	MIC/2	**0.25(32)**	**0.25(16)**	**0.25(32)**	**1(16)**	**0.5(16)**	**0.25(32)**
	MIC/4	**0.25(32)**	**0.25(16)**	**1(8)**	**4(4)**	**2(4)**	**1(8)**
AMP	0	4	4	32	16	8	8
	MIC/2	**0.5(8)**	2(1)	**16(2)**	4(4)	8(1)	**4(2)**
	MIC/4	**0.5(8)**	2(1)	**16(2)**	8(2)	8(1)	**4(2)**
DOX	0	2	8	8	8	16	8
	MIC/2	**0.125(8)**	**0.5(16)**	**2(4)**	**0.5(16)**	**0.5(32)**	**2(4)**
	MIC/4	**0.25(4)**	**1(8)**	**4(2)**	**2(4)**	**0.5(32)**	**4(2)**

Antibiotics tested at 256 μg/mL [STR streptomycin, *ERY* Erythromycin, *NOR* Norfloxacin, *AMP* ampicillin, *CIP* Ciprofloxacin, *DOX* Doxycycline, *CHL* Chloramphenicol, *TET* Tetracycline]. *MIC* Minimal Inhibitory Concentrations; Values in bold represent significant antibacterial activity and significant antibiotic-modulating factor (AMF). AMF ≥2 was set as the cut-off for biologically significance of antibiotic resistance modulating effects [27]

**Table 5:** MICs of antibiotics in association with phytochemical **9** against selected bacteria

Antibiotics	Concentrations of compound 9	Bacterial strain, MIC (μg/mL) and AMF (in bracket)					
		***E. coli***		***P. aeruginosa***		***K. pneumoniae***	
		ATCC10536	AG102	PA01	PA124	ATCC11296	KP55
STR	0	8	16	64	32	16	4
	MIC/2	**0.5(16)**	**8(2)**	**16(4)**	16(2)	**32(2)**	**2(2)**
	MIC/4	**2(4)**	**16(4)**	**32(2)**	32(1)	**32(2)**	**2(2)**
ERY	0	8	4	16	16	32	8
	MIC/2	4(2)	**1(4)**	4(4)	**8(2)**	32(1)	**4(2)**
	MIC/4	4(2)	**0.5(8)**	8(2)	**16(1)**	32(1)	8(1)
NOR	0	32	2	16	8	32	16
	MIC/2	**16(2)**	**0.25(8)**	**4(4)**	**2(4)**	**2(16)**	**4(4)**
	MIC/4	**8(4)**	**0.5(4)**	16(1)	**2(4**)	**8(8)**	**4(4)**
CIP	0	8	4	8	16	8	8
	MIC/2	**0.5(16)**	**0.5(8)**	**0.25(32)**	**0.5(32)**	**0.5(16)**	**1(8)**
	MIC/4	**1(8)**	**2(2)**	**0.5(16)**	**1(16)**	**0.5(16)**	**2(4)**
AMP	0	4	4	32	16	8	8
	MIC/2	**0.5(8)**	2(1)	**16(2)**	**4(4)**	8(1)	**2(4)**
	MIC/4	**1(4)**	2(1)	**16(2)**	**8(2)**	8(1)	**4(2)**
DOX	0	2	8	8	8	16	8
	MIC/2	**0.125(16)**	**0.5(16)**	**1(8)**	**0.5(16)**	**0.5(32)**	**1(8)**
	MIC/4	**0.25(4)**	**1(8)**	**4(2)**	**2(4)**	**0.5(32)**	**4(2)**

Antibiotics tested at 256 μg/mL [STR streptomycin, *ERY* Erythromycin, *NOR* Norfloxacin, *AMP* ampicillin, *CIP* Ciprofloxacin, *DOX* Doxycycline, *CHL* Chloramphenicol, *TET* Tetracycline]. *MIC* Minimal Inhibitory Concentrations; Values in bold represent significant antibacterial activity and significant antibiotic-modulating factor (AMF). AMF ≥2 was set as the cut-off for biologically significance of antibiotic resistance modulating effects [27]

### Effect of compound **7** and **8** on proton-ATPase pumps

**Fig. 2:**
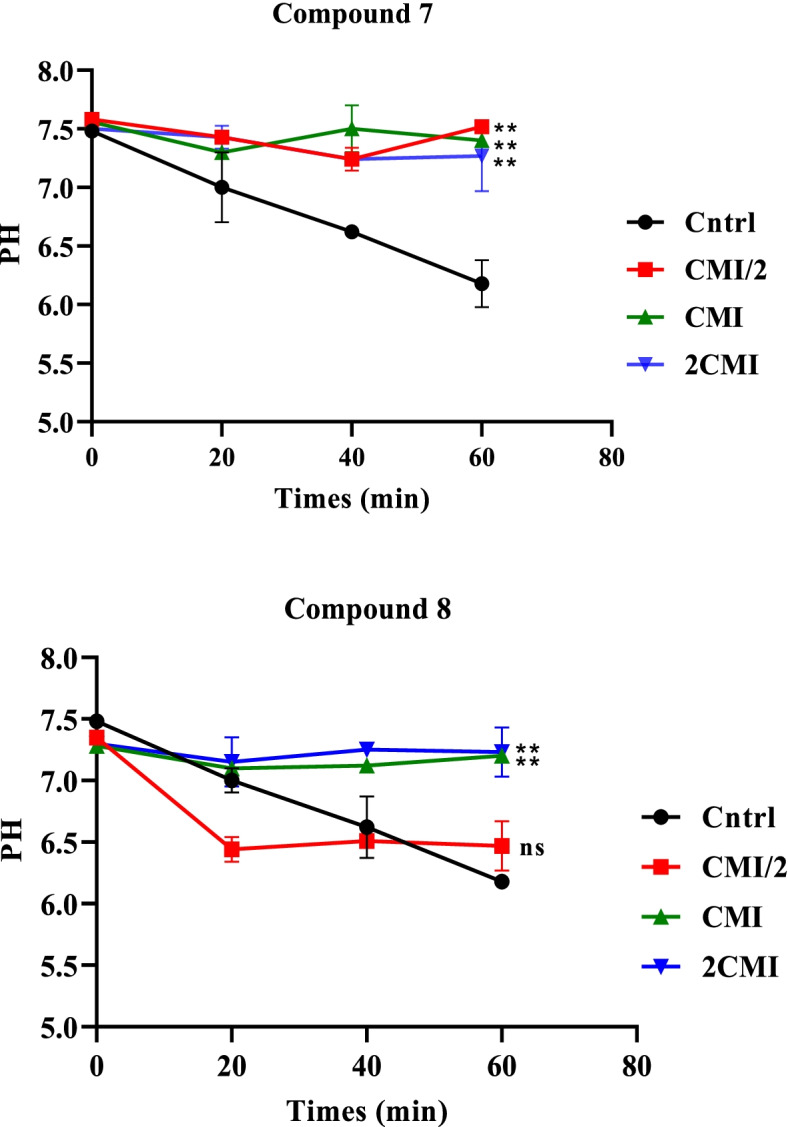
**(a)** Effects of compound 7 and (**b**) compound 8 from *D. welwitschia* on ATPase pumps of *E. coli* AG102. MIC: Minimum inhibitory concentration. Results are expressed as Mean ± SD. Significantly different from the control, * *p* < 0.05, ** *p-values* < 0.01, and *** *p-values* < 0.001; ns: non-significant

Figure 2a and b display the effects compounds **7** and **8** on the activity of proton-ATPase pumps. The differences observed in compounds 7 and 8 tested at MIC/2, MIC and 2MIC were significant compared to the control. In compound 7, a slight decrease in pH was observed, reaching 7.35 at a time equal to 20 min. Then a constant progression of pH to 7.35 until a time t = 60 min overall, with small variations of pH between 7.3 and 7.45 during the experiment, at concentrations equal to MIC/2, MIC and 2MIC. Contrary to the control whose pH decreases from the beginning of the experiment until reaching pH 6.2 at the end (T = 60 min) (Fig. 2a). In compound 8, a constant progression of pH (pH 7.3) can be observed until the end of the experiment, at MIC and 2MIC concentrations. At CMI/2, a decrease in pH is observed until reaching 6.4 at t = 20 min, then remains constant until the end of experiment. Contrary to the control whose pH decreases from the beginning of the experiment until reaching pH 6.2 at the end (t = 60 min) (Fig. 2b).

### Effect of compound **7** and **8** on cellular ATP production

**Fig. 3:**
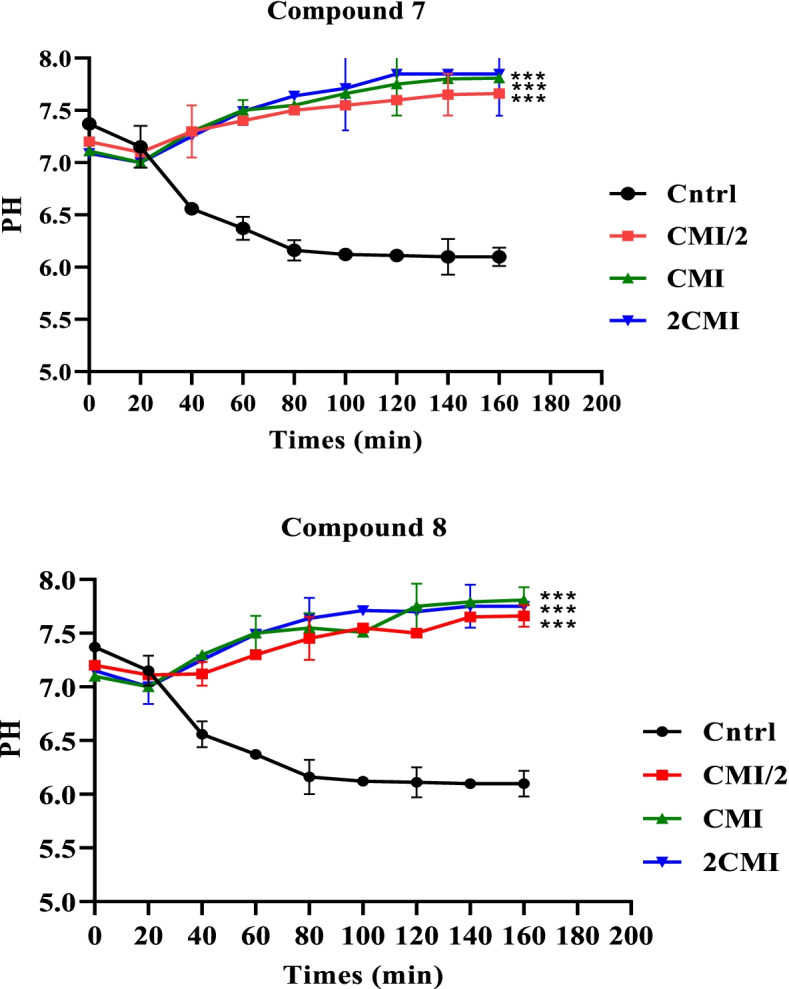
**(a)** Effects of compound 7 and (**b**) compound 8 from *D. welwitschia* on cellular ATP production in *E. coli* AG102. MIC: Minimum inhibitory concentration. Results are expressed as Mean ± SD. Significantly different from the control, * *p* < 0.05, ** *p-values* < 0.01, and *** *p-values* < 0.001; ns: non-significant

Figure 3a and b show the results of the effects of actives compounds **7** and **8** on the activity of ATP synthase. The differences observed in compounds 7 and 8 tested at MIC/2, MIC and 2MIC were significant compared to the control. For compound 7, an increase in pH can be observed from the second minute of the experiment (pH 7.9), at concentrations of MIC/2, MIC and 2MIC. Contrary to the control whose pH decreases from the beginning of the experiment until reaching pH 6.1 at the end (T = 160 min) (Fig. 3a). Virtually the same behaviour was observed with compound 8 (Fig. 3b).

## Discussion

Chemical compounds produced by plants, generally help them to resist fungi, bacteria, and plant virus infections, and consumption by insects and other animals. However, some phytochemicals’ class have been used as medicine to cure various ailments. In the current work, we explored the anti-MDR bacteria’s chemicals of *Donella welwitschii,* as well as the mechanism of action through which they exhibited their antibacterial effects. The chemical investigation of the methylene chloride-methanolic extract of *D. welwitschii* aerial part, led to isolation of eleven compounds belonging to various class of secondary metabolites known for their antimicrobial activities through specific mechanisms of actions i.e. terpenoids, phenolic compounds and alkaloids [29, 30]. Vanillic acid **3** [31] is a phenolic acid belonging to phenolic compounds’ group, taraxerol **4** [32], taraxeryl acetate **5** [33], ursolic acid **6** [34], diospyric acid **7** [35], 28-hydroxy-*β*-amyrin **8** [36] and Oleanolic acid **9** [37] are all triterpenes belonging to terpenoids group**.**

Against a panel of MDR Gram-negative and Gram-positive bacteria, *D. welwitschii’s* compounds **5** and **11** were found to be inactive, while other compounds (**1–4, 6–10**) were selectively significantly active, moderately active and/or weakly active. Diospyric acid (**7**) significantly inhibited (MIC < 10 μg/mL) the growth of *E. coli* AG102, *E. aerogenes* ATCC 13048, *K. pneumoniae* KP55, *P. stuartii* NEA16 and *S. aureus* MRSA3. To the best of our knowledge, this study is illuminating for the first time the antibacterial activity of diospyric acid against the Gram-negative and Gram-positive MDR bacteria. The significant activity of 28-hydroxy-*β*-amyrin toward *E. aerogenes* EA27, *K. pneumonia* ATCC11296 and *S. aureus* MRSA6 is in accordance with the study of Abdel-Raouf et al. [38] which highlighted the interesting inhibition growth capacity of *β*-amyrin against *Staphylococcus aureus* NCTC 7447 and *Salmonella typhi* ATCC 19430. Oleanolic acid (**9**), one of the isolated triterpenes compounds, significantly impaired the growth of *E. coli* AG102, *E. aerogenes* EA27 and *P. stuartii* PS2636). The antimicrobial activity and mechanism of action of oleanolic acid is widely reported in the literature. Martins et al. [39] showed the antibacterial activity of oleanolic against *E. coli* AG100, Methicillin Resistant *Staphyloccocus aureus* COL, *S. aureus* HPH 107, and *Mycobacterium tuberculosis* H37Rv. Moreover, Fontanay et al. [40] showed the effect of oleanolic acid against *E. faecalis* ATCC 28212. In the present work, we also investigated the effect of diospyric acid (7) and 28-hydroxy-*β*-amyrin (**8)** on the functioning of H^+^-ATPase pump and the cellular ATP production of *E. coli* AG102. Let’s note that from the studies of Futai and Kanazawa [41] and those of Senior and Wise [42], it is well established that the H + -ATPase pump of *Escherichia coli* membranes catalyses ATP synthesis and the formation of ATP-driven proton electrochemical gradients, and resembles analogous enzymes from mitochondria, chloroplasts, and other bacteria. The *D. welwitschii*’s compounds might induce bacteria growth inhibition through significant perturbation of H^+^-ATPase pump and inhibition of the cellular production of ATP.

Clinically tripartite efflux systems, AcrAB-TolC for *Enterobacteriaceae* or MexAB-OprM for *P. aeruginosa*, are associated with multidrug resistance of pathogenic Gram-negative bacteria [43–45]. PAßN has been reported as a potent inhibitor of the RND efflux systems and is especially active on AcrAB-TolC and MexAB-OprM [46–49]. PAßN has been used to demonstrate the role of efflux pumps in MDR of the tested microorganisms. In presence of the efflux pump inhibitor PAβN, the MIC values of phytochemicals **7–9** decreased, showing that these compounds could be substrates of efflux pumps acting in resistant strains used in our study. These data suggested that combination of these compounds with antibiotics could improve the antibacterial activity of conventional antibiotics against MDR phenotypes. Combining phytoconstituents **7–9** with antibiotics against MDR bacteria at MIC/2 and MIC/4 strongly improved the antibacterial activity of norfloxacin, ciprofloxacin and doxycycline with antibiotic-modulating factors ranging between 2 and 64. This indicated that compounds **7**, **8** and **9** behave as natural efflux pumps inhibitor. Martins et al. [39] revealed the antibiotic potentiating capacity (tetracycline) of *β*-amyrin (**8)** and Oleanolic acid **(9)** against *E. coli* AG100_TET8_*.* Moreover, our results are in accordance with those of Abreu et al.[50] which highlighted synergism effect against drug-resistant *Staphylococcus aureus* when oleanolic acid was combined to tetracycline or erythromycin, as well as those of Basri et al.[51] showing the synergistic interaction arising from the combination of the oleanolic acid and norfloxacin against SA1199B and MRSA274829 [51] .

## Conclusion

The overall results of the current work provide baseline information for the possible use of some compounds, diospyric acid (**7**), 28-hydroxy-*β*-amyrin (**8**) and Oleanolic acid (**9**), of *Donella welwitschii* extract to fight against bacterial infections involving MDR phenotypes. In addition, the data reported herein indicated that compounds diospyric acid (**7**), 28-hydroxy-*β*-amyrin (**8**) and Oleanolic acid (**9**) behave like efflux pump inhibitors. They are the major antibacterial constituents of *D. welwitschii* against MDR Gram-negative bacteria as well as *Staphylococcus aureus*.

## Supplementary Information

**Additional file 1. SM1**. Physical properties and NMR data of Compounds 1–10. SM2. **Table S1:** Bacterial features of the tested of microorganisms.

## Data Availability

All data generated or analysed during this study are included in this published article and the supporting file.

## Abbreviations

ATCC: American-type culture collection
ATP: Adenosine triphosphate
CFU: Colony-forming unit
CIP: Ciprofloxacin
DMSO: Dimethyl sulfoxide
EPIs: Efflux pump inhibitors
INT: *P*-Iodonitrotetrazolium chloride
MBC: Minimal bactericidal concentration
MDR: Multidrug resistance
MHA: Mueller–Hilton agar
MHB: Mueller–Hinton broth
MIC: Minimal inhibitory concentration
MRSA: Methicillin-resistant *Staphylococcus aureus*
NMR: Nuclear magnetic resonance
PaβN: Phenylalanine arginine-β- naphthylamide
TLC: Thin-layer chromatography
