# Supplementary material for: Antibacterial and antibiotic-potentiation activity of the constituents from aerial part of Donella welwitshii (Sapotaceae) against multidrug resistant phenotypes
Source: BMC Complement Med Ther. 2022 Jul 20;22:194. doi: 10.1186/s12906-022-03673-3 (PMC9301822; doi:10.1186/s12906-022-03673-3)
Supplement: Supplementary file 1 — Additional file 1. SM1. Physical properties and NMR data of Compounds 1–10. SM2. Table S1: Bacterial features of the tested of microorganisms. [file 12906_2022_3673_MOESM1_ESM.docx]

Antibacterial and antibiotic-potentiation activity of the constituents from aerial part of *Donella welwitshii* (Sapotaceae) against multidrug resistant phenotypes

Michel-Gael F. Guefack^1^, Marcelle O. Ngangoue^2^, Armelle T. Mbaveng^1^, Paul Nayim^1*^, Carine M. N. Ngaffo^1^, Jenifer R. N. Kuete^3^, Godloves F. Chi^4^, Bathelemy Ngameni^5^, Bonaventure T. Ngadjui^2^, Victor Kuete^1^*

^1^*Department of Biochemistry, Faculty of Science, University of Dschang, Dschang, Cameroon;*

^2^ *Department of Organic Chemistry, Faculty of Science University of Yaoundé 1, Yaoundé, Cameroon*

*^3^* *Department of Chemistry, Faculty of Science, University of Dschang, Dschang, Cameroon*

*^4^* *Department of Chemistry, Faculty of Science, University of Buea, Buea, Cameroon*

*^5^Department of Pharmacognosy and Pharmaceutical Chemistry, Faculty of Medicine and Biomedical Sciences, University of Yaoundé I, Yaoundé, Cameroon*

*****Corresponding author:**

*E-mail:* [*armbatsa@yahoo.fr*](mailto:armbatsa@yahoo.fr)*; ORCID:* *0000-0003-4178-4967 (Prof. Dr. Armelle T. Mbaveng); E-mail:* [*kuetevictor@yahoo.fr*](mailto:kuetevictor@yahoo.fr)*; ORCID:* *0000-0002-1070-1236 (Prof. Dr. Victor Kuete)*

*Other authors emails:*

*Michel-Gael F. Guefack:* [*michelfofack@gmail.com*](mailto:michelfofack@gmail.com)

*Marcelle O. Ngangoue:* [*marcellengangoue@yahoo.com*](mailto:marcellengangoue@yahoo.com)

*Paul Nayim:* [*nayimpaul@yahoo.fr*](mailto:nayimpaul@yahoo.fr)

*Carine M. N. Ngaffo:* [*ngaffocarine993@gmail.com*](mailto:ngaffocarine993@gmail.com)

*Jenifer R. N. Kuete:* *[jeniferkuete@gmail.com](mailto:jeniferkuete@gmail.com)*

*Godloves F. Chi:* [*chigodloves@yahoo.com*](mailto:chigodloves@yahoo.com)

*Bathelemy Ngameni:* [*bath_ngameni@yahoo.fr*](mailto:bath_ngameni@yahoo.fr)

*Bonaventure T. Ngadjui:* [*ngadjuibt@yahoo.fr*](mailto:ngadjuibt@yahoo.fr)

***SM1. Physical properties and NMR data of Compounds 1 – 11.***

Compound (**1**): 3-*β*-D-glucopyranosyl-2,4,6-trihydroxyl(4-phenyl)methanone**,** (C_19_H_20_O_10_); red-brown paste soluble in MeOH, HRESI-MS [M+H]^+^ at m/z: 410.1210.^1^H NMR (400 MHz, DMSO),  (ppm): 7.58 (2H, d, J = 8.8 Hz, H-2’, H-6’), 6.79 (2H, d, J = 8.4 Hz, H-3’, H-5’), 5.96 (1H, s, H-5), 4.61 (1H, d, J = 9.6 Hz, H-1’’), 3.64 (1H, d, J = 9.2 Hz, H-2’’), 3.58 (1H, d, J = 13.8 Hz, H-3’’), 3.51 (1H, dd, J = 4.5 Hz, H-4’’), 3.20 (2H, dd, J = 6.6 Hz, H-6’’), 3.19 (1H, m, J = 16.5 Hz, H-5’’). ^13^C NMR (100 MHz, DMSO),  (ppm): 195.1 (C-7), 161.8 (C-4’), 159.5 (C-4), 157.9 (C-3), 157.2 (C-2), 132.0 (C-2’, C-6’), 131.2 (C-1’), 115.1 (C-3’, C-5’), 107.4 (C-1), 104.1 (C-5), 95.3 (C-5), 81.6 (C-5’’), 78.8 (C-3’’), 75.1 (C-1’’), 72.3 (C-2’’), 70.1 (C-4’’), 61.0 (C-6’’) [1].

Figure S1: Figure S1: ^1^H NMR spectrum of compound 1

Figure S2: ^13^C NMR spectrum of compound 1

Figure S3: COSY NMR spectrum of compound 1.

Figure S4: HSQC spectrum of compound 1.

Figure S5: HMBC spectrum of compound 1.

Figure S6: HRESI-Mass Spectrum of compound 1.

Figure S7: Chemical Dichroism spectrum of compound 1.

Compound (**2**): 3-*α*-D-glucopyranosyl-2,4,6-trihydroxyl(4-phenyl)methanone (C_19_H_20_O_10_); red-brown paste soluble in MeOH, HRESI-MS [M+H]^+^ at m/z: 433.1112. ^1^H NMR (400 MHz, DMSO),  (ppm): 7.58 (2H, d, J = 8.4 Hz, H-2’, H-6’), 6.79 (2H, d, J = 8.4 Hz, H-3’, H-5’), 5.96 (1H, s, H-5), 4.78 (1H, d, J = 5.6 Hz, H-1’’), 4.60 (1H, t, J = 5.2, 10.0 Hz, H-2’’), 3.64 (1H, dd, J = 5.6, 11.6 Hz, H-3’’), 3.59 (1H, dd, J = 3.6, 5.2 Hz, H-4’’), 3.50 (2H, dd, J = 6.4 Hz, H-6’’), 3.20 (1H, *br*s, H-5’’). ^13^C NMR (100 MHz, DMSO),  (ppm): 195.1 (C-7), 161.8 (C-4’), 159.5 (C-4), 157.8 (C-3), 157.2 (C-2), 132.0 (C-2’, C-6’), 131.2 (C-1’), 115.1 (C-3’, C-5’), 107.4 (C-1), 104.1 (C-5), 95.3 (C-5’), 81.5 (C-5’’), 78.8 (C-3’’), 75.1 (C-1’’), 72.3 (C-2’’), 70.1 (C-4’’), 60.9 (C-6’’) [2].

Figure S8: ^1^H NMR spectrum of compound 2

Figure S9: ^13^C NMR spectrum of compound 2.

Figure S10: ^13^C DEPT 135 NMR spectrum of compound 2.

Figure S11: ^13^HSQC spectrum of compound 2

Figure S12: HMBC spectrum of compound 2.

Figure S14: HR-ESI-Mass Spectrum of compound 2.

Figure S15: Chemical Dichroism Spectrum of compound 2.

Compound (**3**): 4-hydroxy-3-methoxybenzoic acid or vanillic acid (C_8_H_8_O_4_); yellow solid soluble in pyridine. ^1^H NMR (600 MHz, C_5_D_5_N),  (ppm): 8.15 (1H, dd, J = 8.7, 2.4 Hz, H-6), 8.10 (1H, d, J = 8.7 Hz, H-2), 7.31 (1H, d, J = 8.7 Hz, H-5), 3.75 (1H, s, OC**H_3_**). ^13^C NMR (150 MHz, C_5_D_5_N),  (ppm): 169.8 (COOH), 153.3 (C-3), 148.9 (C-4), 125.4 (C-1), 123.1 (C-6), 116.7 (C-2), 114.3 (C-5), 55.6 (O**C**H_3_) [3].


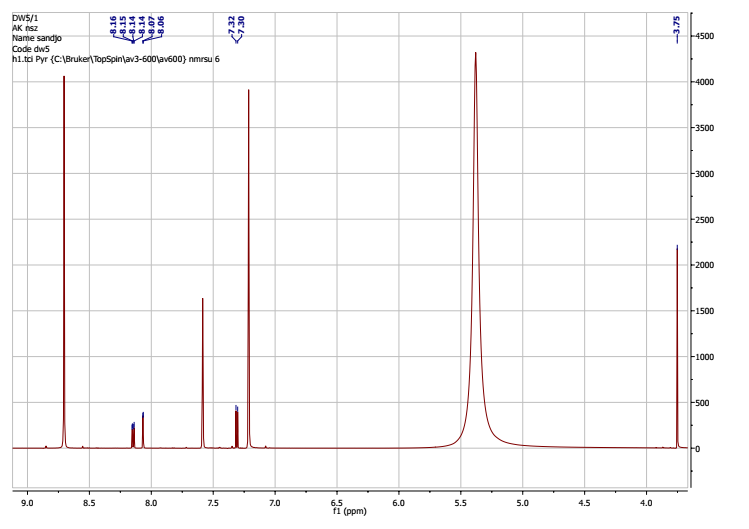


Figure S16: ^1^H NMR of compound 3


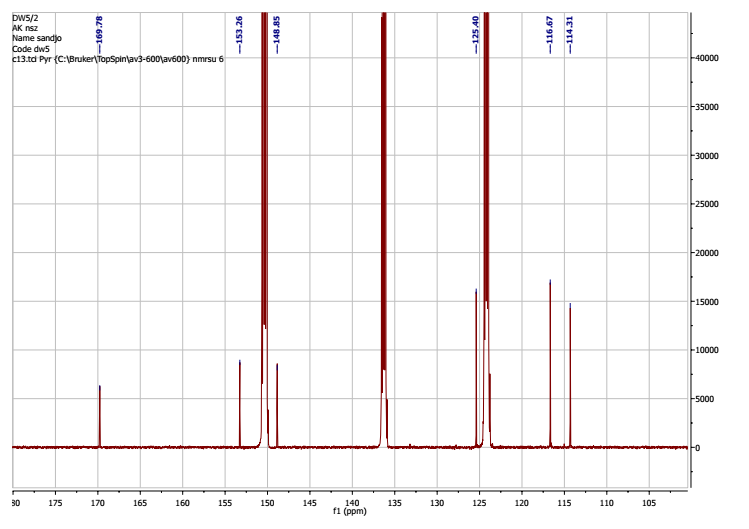


Figure S17: ^13^C NMR of compound 3.

Compound (**4**): Taraxerol (C_30_H_50_O); white powders soluble in dichloromethane. ^1^H-NMR ^13^C (300 MHz, CDCl_3_) δ(ppm): 5.60 (1H, dd, 8.1 and 3.3 Hz, H-15), 3.25 (1H, dd, 10.2 and 4.3 Hz, H-3), 2.10 (1H, dd, 12.6, 3.3 Hz, H-5), 1.99 (1H, dd, 11.7, 3.3 Hz, H-18), 1.80 – 1.32 (), 1.30 (6H, s, H-23), 1.14 (3H, s, H-26), 1.03 (3H, s, H-29), 1.00 (3H, s, H-25), 0.98 (3H, s, H-24), 0.96 (3H, s, H-30), 0.87 (3H, s, H-27), 0.85 (3H, s, H-28). ^13^C-NMR (75 MHz, CDCl_3_) δ (ppm); 158.1 (C-14), 116.9 (C-15), 79.1 (C-3), 55.5 (C-5),49.3 (C-18) ,48.8 (C-9), 41.3 (C-19), 39.0 (C-4), 38.8 (C-8), 38.0 (C-1), 37.8 (C-10), 37.8 (C-17), 37.6 (C-13), 36.7 (C-16), 35.8 (C-12), 35.1 (C-7), 33.7 (C-21) , 33.1 (C-22), 30.0 (C-26), 29.9 (C-28), 29.7 (C-9), 28.0 (C-23), 27.2 (C-2), 25.9 (C-27), 21.4 (C-30), 18.8 (C-6 ), 17.5 (C-11), 15.5 (C-25), 15.5 (C-24) [4].

Figure S18: ^1^H NMR Spectrum of compound 4.

Figure S19: ^13^C NMR Spectrum of compound 4.

Compound (**5**): Taraxeryl acetate (C_32_H_52_O_2_); white powders soluble in dichloromethane. ^1^H-NMR ^13^C (300 MHz, CDCl_3_) δ(ppm): 5.59 (1H, dd, 8.1 and 3.3 Hz, H-15), 4.51 (1H, dd, 10.2 and 4.3 Hz, H-3), 2.09 (3H, s, H-2’), 2.08 (1H, dd, 12.6, 3.3 Hz, H-5), 1.99 (1H, dd, 11.7, 3.3 Hz, H-18), 1.80 – 1.32 (CH_2_ and CH), 1.14 (6H, s, H-23), 1.13 (3H, s, H-26), 1.00 (3H, s, H-29), 0.95 (3H, s, H-25), 0.95 (3H, s, H-24), 0.92 (3H, s, H-30), 0.90 (3H, s, H-27), 0.86 (3H, s, H-28). ^13^C-NMR (75 MHz, CDCl_3_) δ (ppm); 171.0 (C-1’) 158.0 (C-14), 116.9 (C-15), 81.0 (C-3), 55.7 (C-5),49.2 (C-18) ,48.8 (C-9), 41.2 (C-19), 39.0 (C-4), 37.9 (C-8), 37.7 (C-1), 37.6 (C-10), 37.4 (C-17), 36.7 (C-13), 36.7 (C-16), 35.8 (C-12), 35.1 (C-7), 33.7 (C-21) , 33.4 (C-22), 33.1 (C-26), 29.9 (C-28), 29.9 (C-9), 28.8 (C-23), 23.5 (C-2), 21.4 (C-27), 21.3 (C-30), 18.7 (C-6 ), 17.5 (C-11), 16.6 (C-25), 15.5 (C-24) [5].


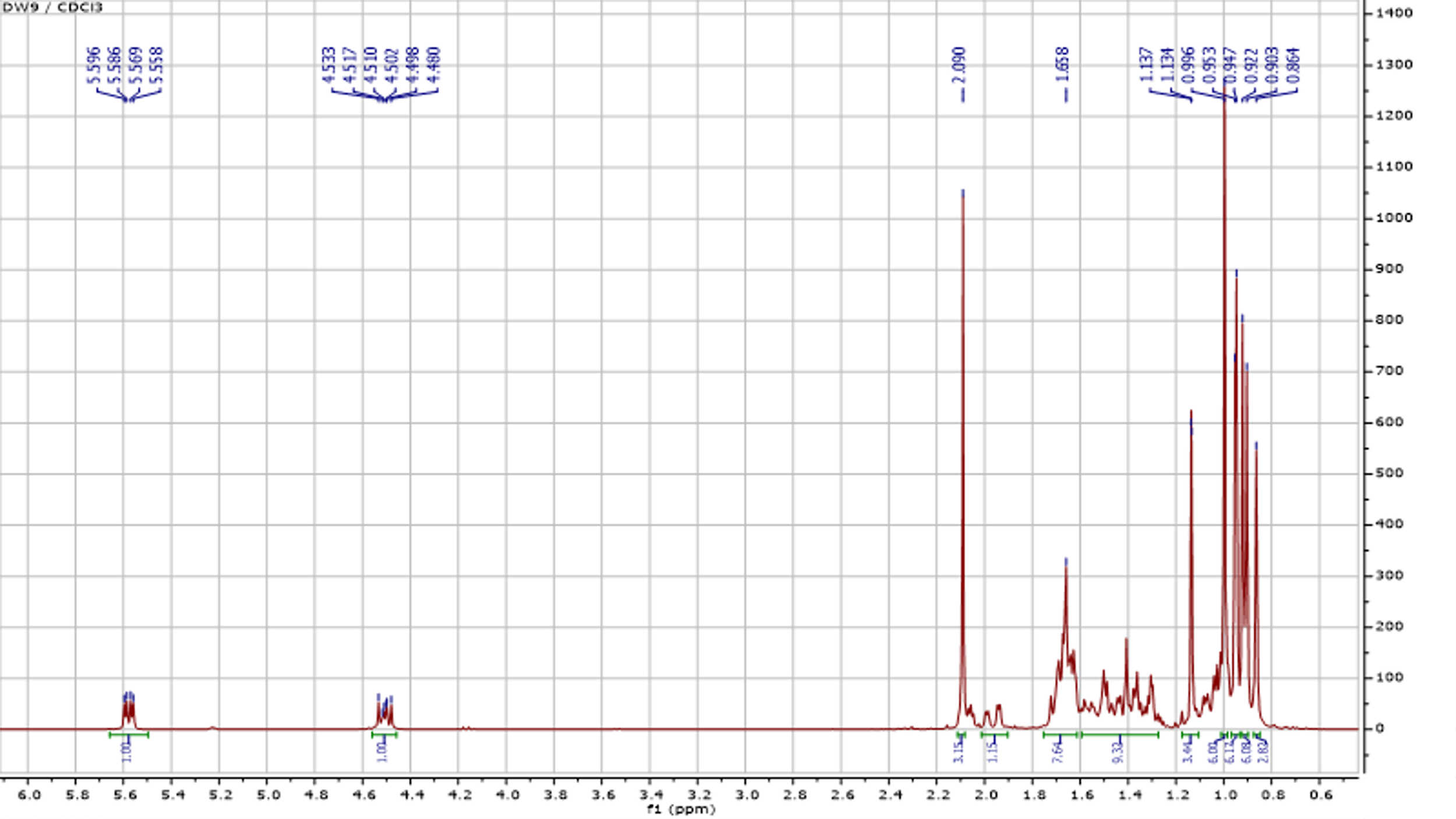


Figure S20: ^1^H NMR Spectrum of compound 5.

Figure S21: ^13^C NMR Spectrum of compound 5.

Figure S22: ^13^C DEPT 135 NMR Spectrum of compound 5.

Compound (**6**): Ursolic acid (C_30_H_50_O_2_): white solid soluble in MeOH, ^1^H NMR (600 MHz, CD_3_OD),  (ppm): 5.28 (1H, t, J = 3.6 Hz, H-12), 3.96 (1H, t, J = 2.9 Hz), 2.57 (1H, dt, J = 4.4 Hz, H-16a), 2.49 (1H, s, H-18), 1.36 (3H, s, H-27), 1.24 (3H, s, H-23), 1.18 (3H, s, H-29), 0.93 (3H, d, J = 6.7 Hz, H-30), 0.88 (3H, s, H-25), 0.81 (3H, s, H-26). ^13^C NMR (CD_3_OD, 150 MHz)  (ppm): 181.6 (C-28), 138.0 (C-13), 126.0 (C-12), 79.2 (C-3), 55.3 (C-5), 52.8 (C-18), 48.0 (C-9), 48.0 (C-17), 48.0 (C-19), 47.6 (C-9), 42.1 (C-14), 39.6 (C-1), 39.6 (C-4), 39.2 (C-20), 38.3 (C-8), 38.7 (C-10), 37.1 (C-22), 37.2 (C-1), 33.5 (C-2), 28.3 (C-2), 25.8 (C-29), 25.7 (C-21), 25.6 (C-11), 25.2 (C-16), 23.7 (C-27), 21.3 (C-23), 19.7 (C-6), 15.7 (C-26), 15.4 (C-30), 12.2 (C-25) [6].


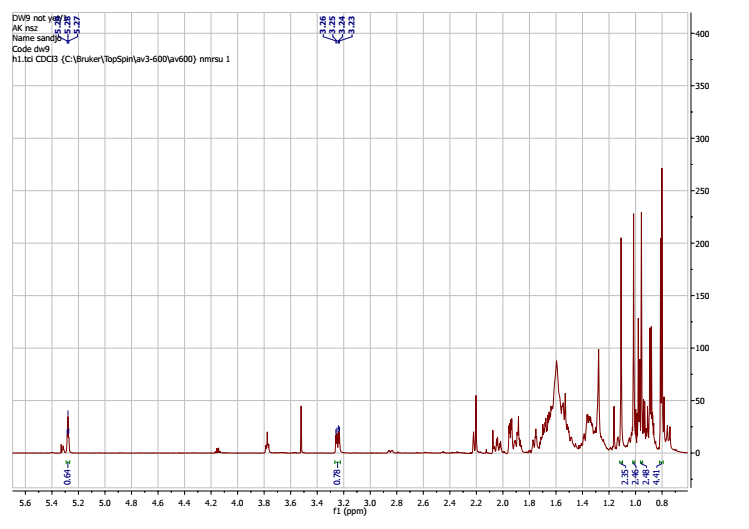


Figure S27: ^1^H NMR Spectrum of compound 6.


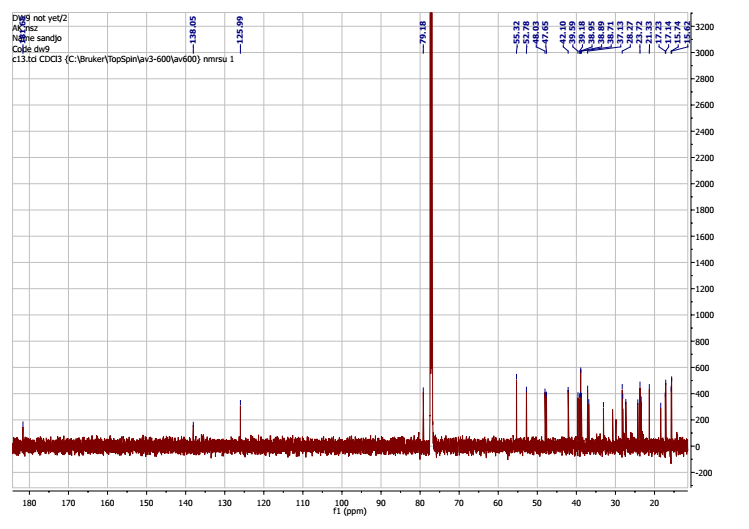


Figure S28: ^13^C NMR Spectrum of compound 6.

Compound (**7**): 3, 19-dihydroxyurs-12-en-24, 28-dioic acid or diospyric acid (C_30_H_46_O_6_): white solid soluble in MeOH, HRESI-MS [M+H]^+^ at m/z: ^1^H NMR (400 MHz, CD_3_OD),  (ppm): 5.28 (1H, t, J = 3.6 Hz, H-12), 3.96 (1H, t, J = 2.9 Hz), 2.57 (1H, dt, J = 4.4 Hz, H-16a), 2.49 (1H, s, H-18), 1.36 (3H, s, H-27), 1.24 (3H, s, H-23), 1.18 (3H, s, H-29), 0.93 (3H, d, J = 6.7 Hz, H-30), 0.88 (3H, s, H-25), 0.81 (3H, s, H-26). ^13^C NMR (CD_3_OD, 100 MHz)  (ppm): 180.9 (C-28), 179.9 (C-24), 138.4 (C-13), 128.2 (C-12), 72.1(C-19), 70.2 (C-3), 53.7 (C-18), 48.7 (C-9), 48.0 (C-17), 47.6 (C-4), 47.0 (C-9), 39.6 (C-8), 37.5 (C-10), 37.2 (C-1), 33.5 (C-2), 28.1 (C-15), 25.8 (C-29), 25.7 (C-21), 25.6 (C-11), 25.2 (C-16), 23.4 (C-27), 23.2 (C-23), 19.7 (C-6), 15.9 (C-26), 15.1 (C-30), 12.2 (C-25) [7].

Figure S23: ^1^H NMR Spectrum of compound **7**.

Figure S24: ^13^C NMR Spectrum of compound **7**.

Compound (**8**): 28-hydroxy-*β*-amyrin (C_30_H_50_O_2_): white solid soluble in MeOH, ^1^H NMR (600 MHz, CD_3_OD),  (ppm): 5.28 (1H, t, J = 3.6 Hz, H-12), 3.96 (1H, t, J = 2.9 Hz), 2.57 (1H, dt, J = 4.4 Hz, H-16a), 2.49 (1H, s, H-18), 1.36 (3H, s, H-27), 1.24 (3H, s, H-23), 1.18 (3H, s, H-29), 0.93 (3H, d, J = 6.7 Hz, H-30), 0.88 (3H, s, H-25), 0.81 (3H, s, H-26). ^13^C NMR (CD_3_OD, 150 MHz)  (ppm): 145.6 (C-13), 123.5 (C-12), 79.7 (C-3), 67.8 (C-28), 56.7 (C-5), 47.8 (C-9), 42.8 (C-18), 41.9 (C-8), 39.9 (C-20), 39.8 (C-19), 38.1 (C-4), 38.0 (C-1), 36.8 (C-10), 33.7 (C-17), 33.7 (C-21), 32.3 (C-16), 32.0 (C-7), 31.8 (C-21), 28.7 (C-2), 26.5 (C-15), 24.6 (C-11), 24.0 (C-27), 23.2 (C-23), 19.7 (C-6), 17.3 (C-26), 16.3 (C-30), 16.0 (C-25) [8].


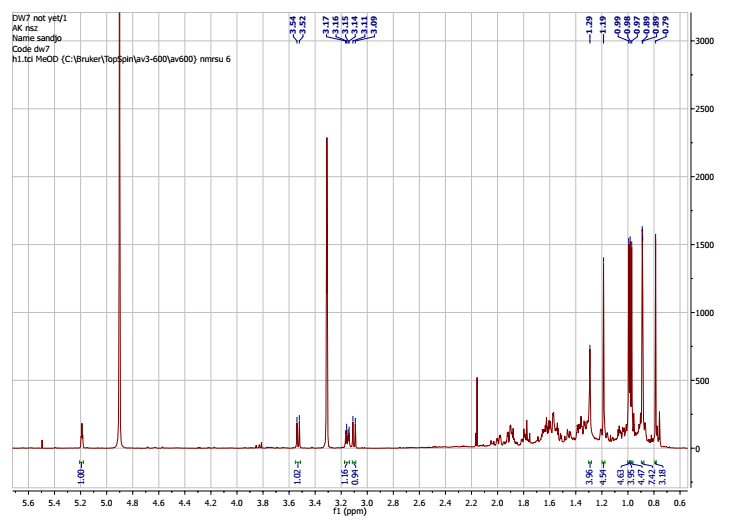


Figure S25: ^1^H NMR Spectrum of compound **8**.


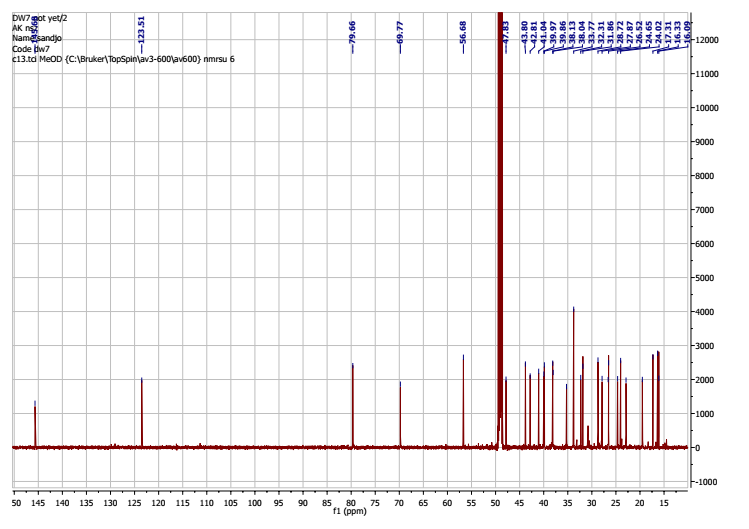


Figure S26: ^13^C NMR Spectrum of compound **8**.

Compound (**9**): Oleanolic acid (C_30_H_50_O_2_): Oleanolic acid (C_30_H_50_O_2_): white solid soluble in CDCl_3_, EI-MS m/z 456.4. ^1^H NMR (400 MHz, CDCl_3_) δ (ppm) 5.27 (1H, t, 4.0 Hz, H-12), 3.20 (1H, dd, 11.0, 5.1 Hz, H-3), 2.80 (1H, dd, 13.6, 4.4 Hz, H-18), 1.97 (1H, td, 4.0, 3.6, H-9), 1.12 (3H, s, H-27), .97 (3H, s, H-23), 0.91 (3H, s, H-26), 0.89 (3H, s, H-24), 0.88 (3H, s, H-25), 0.75 (3H, s, H-30), 0.74 (3H, s, H-29). ^13^C NMR (125 MHz, CDCl_3_)  (ppm): 180.4 (C-28), 145.1 (C-13), 123.2 (C-12), 79.2 (C-3), 55.3 (C-5), 52.9 (C-18), 48.0 (C-17), 47.7 (C-9), 42.8 (C-14), 39.6 (C-8), 39.2 (C-8), 39.0 (C-20), 38.9 (C-4), 37.1 (C-22), 34.5 (C-7), 32.3 (C-28), 30.0 (C-23), 28.3 (C-15), 23.7 (C-11), 21.3 (C-30), 17.2 (C-26), 17.1 (C-29), 15.7 (C-25), 15.6 (C-24) [9].

Figure S29: ^1^H NMR Spectrum of compound 9.

Compound (**10**):* α*-Spinasterol (C_29_H_48_O), white crystalline solid soluble in CDCl_3_, EI-MS m/z 412.4, melting point 168 - 169°C. ^1^H NMR (300 MHz, CDCl_3_) δ 5.10 (1H, *dd*, J= 8.7, 15.0 Hz, H-22), 5.06 (1H, *dd*, J = 8.7, 15.0 Hz, H-22), 4.96 (1H, dd, J = 6.9, 8.4, Hz, H-7), 3.54 (1H, m, J = 4.2, 6.3 Hz, H-3), 1.94 (2H, m, H-), 1.79 – 1.03 (25H, 9 CH_2_ & 7 CH), 0.97 (3H, d, 6.3 Hz,H-21), 0.79 (3H, d, 6.3 Hz, H-27), 0.77 (3H, d, 3.3 Hz, H-26), 0.76 (3H, t, 3.3 Hz, H-29), 0.73 (3H, s, H-19), 0.48 (3H,s, H-18). ^13^C NMR (75 MHz, CDCl_3_) δ 139.59 (C-8), 138.20 (C-22), 129.47(C-23), 117.49 (C-7), 71.08 (C-3), 55.93 (C-17), 55.16 (C-14), 51.28 (C-24), 49.48 (C-9), 43.31 (C-13), 40.86 (C-20), 40.29 (C-5), 39.50 (C-12), 38.01 (C-4), 37.18 (C-1), 34.25 (C-10), 31.91 (C-25), 31.49 (C-2), 29.67 (C-6), 28.54 (C-16), 25.43 (C-28), 23.05 (C-15), 21.58 (C-11), 21.41 (C-21), 21.13 (C-26), 19.03 (C-27), 13.07 (C-19), 12.28 (C-29), 12.08 (C-18) [10].

Figure S31: ^1^H NMR Spectrum of compound 10.

Figure S32: ^13^C NMR Spectrum of compound 10.

Compound (**11**): Spinasteryl-3-*O*-*β*-D-glucopyranoside or Spinasteryl glucoside (C_36_H_60_O_6_), white crystalline solid soluble in C_5_H_5_N, EI-MS m/z 588.4, melting point 168 - 169°C. ^1^H NMR (600 MHz, C_5_D_5_N) δ 5.10 (1H, *dd*, J= 8.7, 15.0 Hz, H-22), 5.06 (1H, *dd*, J = 8.7, 15.0 Hz, H-22), 4.96 (1H, dd, J = 6.9, 8.4, Hz, H-7), 3.54 (1H, m, J = 4.2, 6.3 Hz, H-3), 1.94 (2H, m, H-), 1.79 – 1.03 (25H, 9 CH_2_ & 7 CH), 0.97 (3H, d, 6.3 Hz,H-21), 0.79 (3H, d, 6.3 Hz, H-27), 0.77 (3H, d, 3.3 Hz, H-26), 0.76 (3H, t, 3.3 Hz, H-29), 0.73 (3H, s, H-19), 0.48 (3H,s, H-18). ^13^C NMR (150 MHz, C_5_D_5_N) δ 139.59 (C-8), 138.20 (C-22), 129.47(C-23), 117.49 (C-7), 71.08 (C-3), 55.93 (C-17), 55.16 (C-14), 51.28 (C-24), 49.48 (C-9), 43.31 (C-13), 40.86 (C-20), 40.29 (C-5), 39.50 (C-12), 38.01 (C-4), 37.18 (C-1), 34.25 (C-10), 31.91 (C-25), 31.49 (C-2), 29.67 (C-6), 28.54 (C-16), 25.43 (C-28), 23.05 (C-15), 21.58 (C-11), 21.41 (C-21), 21.13 (C-26), 19.03 (C-27), 13.07 (C-19), 12.28 (C-29), 12.08 (C-18) [11].


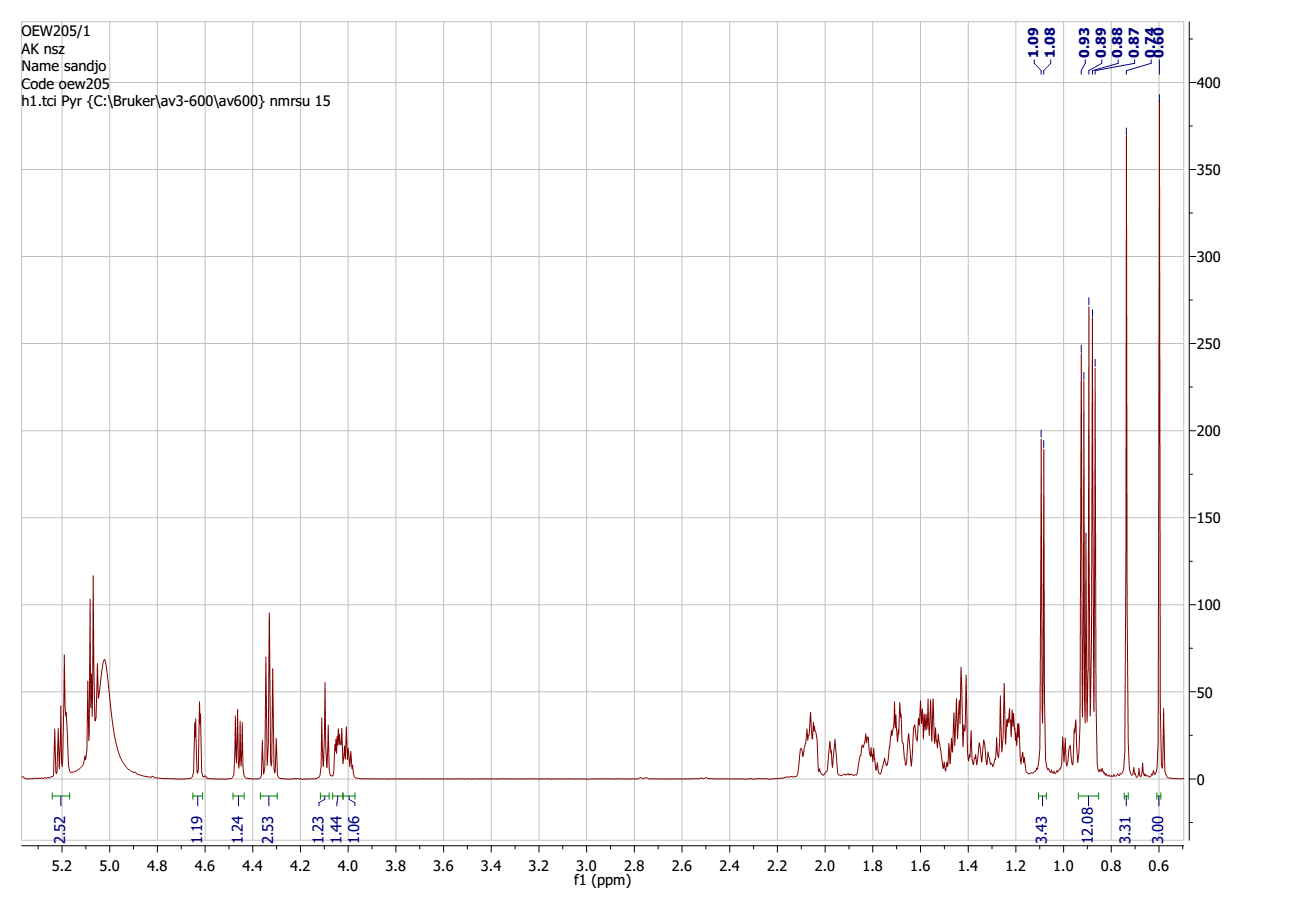


Figure S31: ^1^H NMR Spectrum of compound 11.


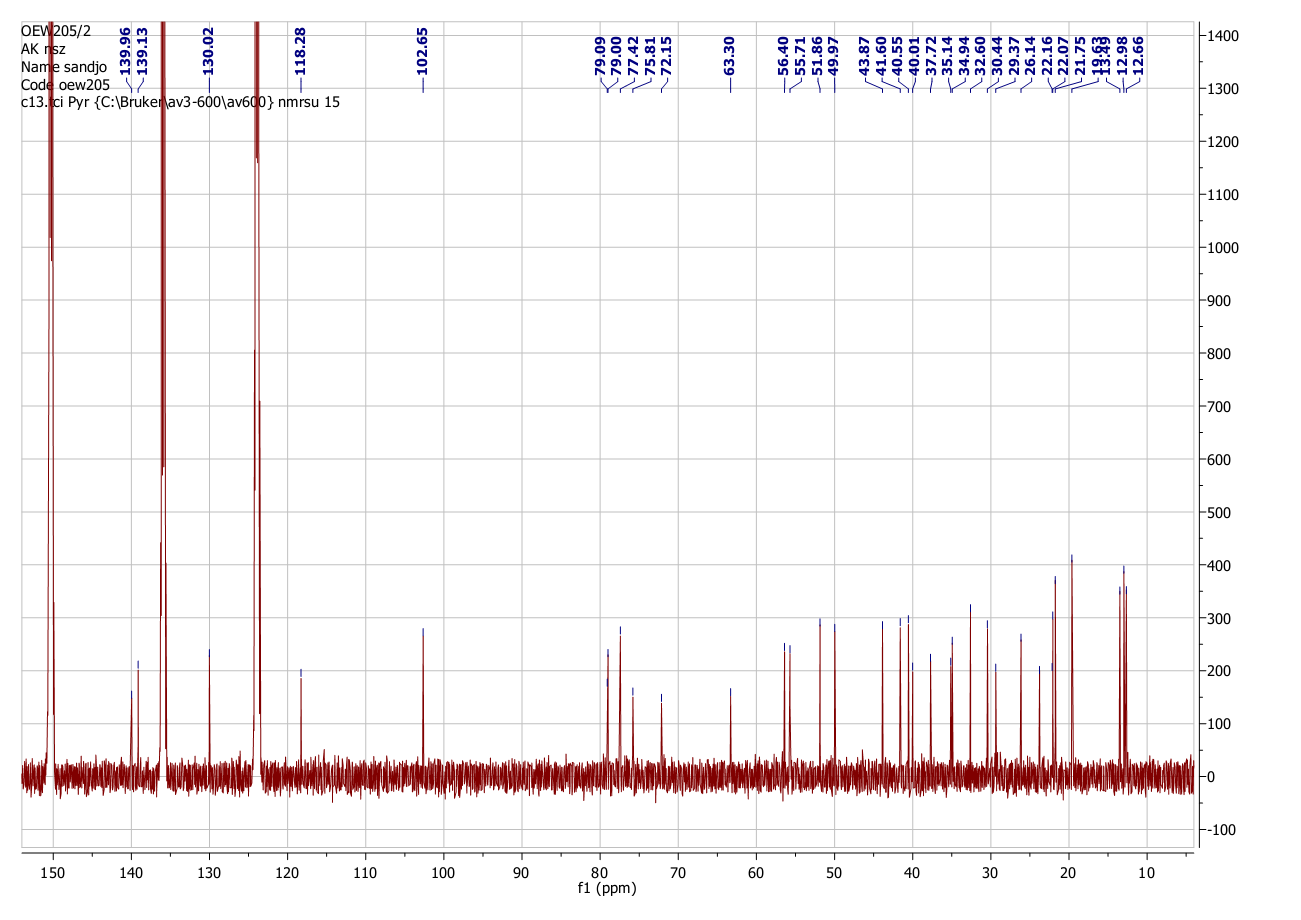


Figure S32: ^13^C NMR Spectrum of compound 11.

***Supplementary material (SM2)***

**Table S1:** Bacterial features of the tested of microorganisms

| Bacteria strains | | Characteristics | References |
| --- | --- | --- | --- |
| *Escherichia*  *coli* | **ATTC10536** | Reference strains | [12] |
|  | **AG102** | AG 100 over expressing *Acr AB* pomps | [12] |
| *Enterobacter aerogenes* | **ATCC13048** | Reference strains | [13, 14] |
|  | **EA 27** | Clinical strain with energy dependent efflux to CHL and NOR; KAN^r^, AMP^r^, NAl^r^, STR^r^, TET^r^ | [13, 14] |
| *Klebsiella pneumoniae* | **ATCC11296** | Reference strains of *Klebsiella pneumoniae* | Collection of the UMR-MD1 laboratory of the University of the Mediterranean, Marseille, France [12]. |
|  | **Kp55** | Clinical strain: TET^r^, AMP^r^, ATM^r^, CEF^r^ |  |
| *Providencia stuartii* | **NEA16** | Clinical isolate of *Providencia stuartii* *AcrAB-TolC* |  |
|  | **PS2636** | *AcrAB-TolcC* associated with porin types OMPF andOMPC |  |
| *Pseudomonas aeruginosa* | **PA01** | Reference strains | Collection of the UMR-MD1 laboratory of the University of the Mediterranean, Marseille, France [12, 15] |
|  | **PA124** | Multi-drug resistant clinical strain *MexAB-OprM* |  |
| *Staphylococcus aureus* | **ATCC25923** | Reference strain | [16, 17] |
|  | **MRSA3** | Clinical isolate: Ofxar, Kanr, Tetr, Ermr |  |
|  | **MRSA6** | Clinical isolate: Ofxar, Flxr, Kanr, Tetr, Cypr, IM/Csr, Chlr, Genr, Nisr, Ampr |  |

AMP^r^, ATM^r^, CEF^r^, CHL^r^, KAN^r^, NAL^r^, NOR^r^, STR^r^ et TET^r^, resistant to: ampicillin, aztreonam, cefepime, chloramphenicol, kanamycin, nalidixic acid, norfloxacin, streptomycin et tetracycline respectively; *AcrAB-TolC, MexAB-OprM :* efflux pomp.

**References**

1. Pan J, Yi X, Wang Y, Chen G, and He X. Benzophenones from Mango Leaves Exhibit ??-Glucosidase and NO Inhibitory Activities. J. Agric. Food Chem. 2016; 64(40), 7475–7480.

2. Suliman SN, Elnaggar MH, Elsbaey M,. El-gamil MM, and Badria FA. Bio-guided Isolation of Natural Iron Chelators from Mangifera indica Leaves and their Comparative Study to Desferal ®. 2021; 27(2), 78–85.

3. Chang SW, Kim KH, Lee IK, Choi SU, Ryu SY, and Lee KR. Phytochemical constituents of Bistorta manshuriensis. Nat. Prod. Sci. 2009; 15(4), 234–240.

4. Sangeetha K, Sujatha S, and Shanmuganathan V. Biochimica et Biophysica Acta 3 β -taraxerol of Mangifera indica , a PI3K dependent dual activator of glucose transport and glycogen synthesis in 3T3-L1 adipocytes. BBA - Gen. Sub*.* 2010; 1800(3). 359–366.

5. Díaz-Ruiz G, Hernández-Vázquez L, Luna H, Del Carmen Wacher-Rodarte M, and Navarro-Ocaña A. Growth inhibition of streptococcus from the oral cavity by α-Amyrin esters. Molecules. 2012; 17(11), 12603–12611.

6. Liu M, Yeng S, Jin L, Hu D, Wu Z, and Yang S. Chemical constituents of the ethyl acetate extract of belamcanda chinensis (L.) DC roots and their antitumor activities. Molecules. 2012; 17(5), 6156–6169.

7. Thuong PT, Lee CH, Dao TT, Nguyen PH, Kim WG, Lee SJ, Oh WK. Triterpenoids from the leaves of Diospyros kaki (persimmon) and their inhibitory effects on protein tyrosine phosphatase 1B. J. Nat. Prod. 2008; 71(10), 1775–1778.

8. S. B. Mahato and Kundu. A. P., “13C Nmr Spectra of Pentacyclic Triterpenoids-a and Some Salient Features,” *Phytochemistry*, 1994; 37, 1517–1575.

9. Seebacher W, Simic N, Weis R, Saf R, and Kunert O. Complete assignments of 1H and 13C NMR resonances of oleanolic acid, 18α-oleanolic acid, ursolic acid and their 11-oxo derivatives. Magn. Reson. Chem*.* 2003; 41(8), 636–638.

10. Ragasa CY, Galian RF, Arenal M, Tan V, and Shen CC. Research Journal of Pharmaceutical , Biological and Chemical Sciences In-vitro Antibacterial and Antifungal Effect of Areca Nut Extract. Res. J. Pharm. Biol. Chem. 2014; 282(7), 282–286.

11. Kim S, Hye Min, Ahn, Mi-Jeong; Lee. Isolation and identification of phytochemical constituents from Scrophularia takesimensis. J. Med. Plants Res. 2012; 22(6), 3923–3930.

12. Chevalier J, Bredin J, Mahamoud A, Mallea M, Barbe J, Pagès J-M. Inhibitors of antibiotic efflux in resistant Enterobacter aerogenes and Klebsiella pneumonia strains. Antimicrob. Agents Chemother. 2004; 48(1), 1043-1046.

13. Ghisalberti D, Masi M, Pagès J-M, Chevalier J. Chloramphenicol and expression of multidrug efflux pump in Enterobacter aerogenes. Biochem. Biophys. Commu. Res. 2005; 328(2), 1113-1118.

14. Malléa M, Chevalier J, Bornet C, Eyraud A, Pagès J-M, Davin-Régli A. Porin alteration and active efflux: two in vivo drug resistance strategies used by Enterobacter aerogenes. Microbiology. 1998, 144 : 3003–3009.

15. Lorenzi V, Muselli A, Bernadini AF, Berti L, Pagès J-M. Geraniol restores antibiotic activities against multidrug resistant isolates from Gram-negatives species. Antimicrob. Agents Chemother. 2009; 53(6), 2209-2211.

16. Paudel A, Hamamoto H, Kobayashi Y, Yokoshima S, Fukuyama T, Sekimizu K. Identification of novel deoxyribofuranosyl indoleantimicrobial agents. J. Antibiotics. 2012; 65(3), 53-57.

17. Dzoyem JP, Hamamoto H, Ngameni B, Ngadjui BT, Sekimizu K. Antimicrobial action mechanism of ﬂavonoids from *Dorstenia* species. Drug Discov. Ther. 2013; 7(1), 66–72.
